# Continuing Professional Development ‐ Radiation Therapy

**DOI:** 10.1002/jmrs.771

**Published:** 2024-02-21

Maximise your CPD by reading the following selected article and answer the five questions. Please remember to self‐claim your CPD and retain your supporting evidence. Answers will be available via the QR code and online at www.asmirt.org/news‐and‐publications/jmrs, as well as published in JMRS – Volume 71, Issue 4, December 2024.

## Radiation Therapy — Original Article

### Dosimetric comparison of gantry and horizontal fixed‐beam proton therapy treatment plans for base of skull chordoma

Shierlaw E, Penfold M, Crain R, Santos AMC, Penfold SN. (2024). *J Med Radiat Sci*. https://doi.org/10.1002/jmrs.742

1. What will be the arrangement of the proton beam therapy (PBT) treatment rooms at the first PBT centre in Australia?
  - 1 fixed beam and 2 gantry
  - 2 fixed beam and 1 gantry
  - 2 fixed beam and 2 gantry
  - 1 fixed beam and 1 gantry
2. What geometric uncertainty was used in the robust optimisation and evaluation of the plans included in this study?
  - 1 mm
  - 2 mm
  - 3 mm
  - 4 mm
3. What was the most common dose prescription for patients included in this study?
  - 70 Gy in 35 fractions
  - 74 Gy in 37 fractions
  - 66 Gy in 33 fractions
  - 72 Gy in 40 fractions
4. In this study, what was the organ at risk planning dose‐volume metric used for the optic nerves?
  - D_2cc_ < 60 Gy
  - D_2%_ < 50 Gy
  - D_0.03cc_ < 60 Gy
  - D_2%_ < 60 Gy
5. According to this study, which organs are classified as primary organs at risk?
  - Brainstem, lens, spinal cord
  - Optic nerve, optic chiasm, brainstem
  - Optic nerve, cochlea, optic chiasm
  - Optic chiasm, brainstem, temporal lobe

### Recommended further reading

1. Palm RF, Oliver DE, Yang GQ, Abuodeh Y, Naghavi AO, Johnstone PAS. The role of dose escalation and proton therapy in perioperative or definitive treatment of chondrosarcoma and chordoma: an analysis of the National Cancer Data Base. *Cancer* 2019; **125**(4): 642–51. https://doi.org/10.1002/cncr.31958
2. Korevaar EW, Habraken SJM, Scandurra D. Practical robustness evaluation in radiotherapy – a photon and proton‐proof alternative to PTV‐based plan evaluation. *Radiother Oncol* 2019; **141**: 267–74. 10.1016/j.radonc.2019.08.005 31492443

## Answers

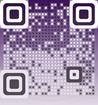

Scan this QR code to find the answers, or visit www.asmirt.org/news‐and‐publications/jmrs

